# Prediction of disease-related mutations affecting protein localization

**DOI:** 10.1186/1471-2164-10-122

**Published:** 2009-03-23

**Authors:** Kirsti Laurila, Mauno Vihinen

**Affiliations:** 1 Institute of Medical Technology, FI-33014 University of Tampere, Finland; 2 Department of Signal Processing, Tampere University of Technology, P.O. Box 527, FI-33101 Tampere, Finland; 3 Tampere University Hospital, FI-33520 Tampere, Finland

## Abstract

**Background:**

Eukaryotic cells contain numerous compartments, which have different protein constituents. Proteins are typically directed to compartments by short peptide sequences that act as targeting signals. Translocation to the proper compartment allows a protein to form the necessary interactions with its partners and take part in biological networks such as signalling and metabolic pathways. If a protein is not transported to the correct intracellular compartment either the reaction performed or information carried by the protein does not reach the proper site, causing either inactivation of central reactions or misregulation of signalling cascades, or the mislocalized active protein has harmful effects by acting in the wrong place.

**Results:**

Numerous methods have been developed to predict protein subcellular localization with quite high accuracy. We applied bioinformatics methods to investigate the effects of known disease-related mutations on protein targeting and localization by analyzing over 22,000 missense mutations in more than 1,500 proteins with two complementary prediction approaches. Several hundred putative localization affecting mutations were identified and investigated statistically.

**Conclusion:**

Although alterations to localization signals are rare, these effects should be taken into account when analyzing the consequences of disease-related mutations.

## Background

Eukaryotic cells contain numerous compartments, such as cytoplasm, mitochondria, Golgi apparatus, and peroxisomes, all of which contain different protein constituents and have different functions. Proteins are typically directed to these compartments by short peptide sequences that act as targeting signals. For example, secretory, chloroplast and mitochondrial targeting peptides are located at the N terminus, whereas signals for other compartments can be within the amino acid sequence. Terminal signal peptides are typically cleaved during the protein translocation process.

Protein function depends on numerous factors. One important but often neglected property is its subcellular localization. Translocation to the proper compartment allows a protein to form the necessary interactions with its partners and take part in biological networks. For example, signalling and metabolic pathways are dependent on the location of the constituent proteins. Failure to be transported to the correct intracellular compartment can have detrimental effects, which appear in different ways. Either the reaction performed or information carried by the protein does not reach the proper site, causing either inactivation of central reactions or misregulation of, eg, signalling cascades, or the mislocalized protein is active, but has harmful effects by acting in the wrong place.

Subcellular localization of proteins and peptides has long been investigated using numerous methods. Recently, high-throughput methods have been developed based either on the use of reporter genes/tags or by purification, fractionation and analysis of cellular compartments [1,2]. Information on protein localization is scattered throughout publications and numerous databases. Fortunately, central resources such as the Human Protein Reference Database (HPRD) [3], UniProt [4] and Gene Ontologies [5] now exist to integrate information from several sources. A problem with these databases, however, is that data quality and experimental methods vary. Further, some databases contain experimentally validated localization information whereas others also contain localization predictions. The picture is further complicated by the fact that a protein can be localized in more than one compartment, often depending on the state of the cell. Thus, databases that contain only experimentally validated data may not provide complete information for all proteins.

Numerous methods have been developed to predict protein subcellular localization (for review, see eg, [6]). The very first methods in the 1970's were developed to identify microbial signal peptides [7,8]. Now, methods and protocols exist for the prediction of over 10 cellular compartments and subcompartments. Although the actual prediction algorithms and methods differ, all are based on sequence signature patterns. Some general predictors are useful for all subcompartments, but the majority of methods are specific for individual compartments and organisms or groups of organisms. The reliability of individual methods is relatively high, close to 90% (see, eg, [9-11])

Disease-causing mutations result in abnormal cellular function through numerous mechanisms. To date, pathological mechanisms have been revealed for only a fraction of all known mutations. Mutation information has been collected and stored in locus-specific (eg, [12,13]) and general (such as Online Mendelian Inheritance in Man (OMIM) and Human Gene Mutation Database (HGMD)) databases. Many experimental methods are tedious, expensive and difficult to use. Disease-causing mutations are identified for diagnostic purposes, and thus most medical centers identify a genetic mutation(s) without acquiring further information about the protein. We and others have applied numerous bioinformatic methods to predict and explain the consequences of mutations. Recently, we discussed the applicability of some 40 analysis and prediction methods [14,15]. The effects and consequences vary depending on the site and type of mutation, with insertions and deletions usually leading to truncated proteins. These cases are easy to explain if a substantial part of the protein is missing. To understand protein structure and function, however, missense mutations are most interesting because they often indicate residues that are critical for, and changes that are deleterious to, structure and/or function. Most mutations reduce protein activity, but increasing numbers of gain-of-function mutations [16,17] are also being identified. Relatively few detailed investigations have described protein mislocalization due to disease-related mutations or introduced genetic alterations. In addition, all such publications report a limited number of mutations in a single protein.

Targeting signals tend to be conserved and thus sensitive to alterations; therefore, we can assume that these methods can be applied to the analysis of point mutations. Here we use bioinformatics to investigate the effects of known disease-related mutations on protein targeting and localization by analyzing 22,416 missense mutations. Several hundred putative localization mutations were identified with two complementary multiprediction approaches. The results indicate that although alterations to localization signals are rare, localization predictors should be added to the methods arsenal of a mutations analyst. Our results also suggest pathological mechanisms for a number of mutations and depict cases for further experimental investigation.

## Results and discussion

We investigated the effects of disease-related mutations on protein localization by performing large-scale analysis and prediction with two different but complementary methods. Because we needed unambiguous mapping of DNA mutations to protein sequences, we performed filtering steps. We obtained experimentally identified protein localizations from HPRD [3], which is considered a highly accurate, consistent and reliable source of protein annotations.

### Reliability of the individual localization predictors

Before approaching the mutation effect predictions, we wanted to test the applicability of the methods to the dataset. Because HPRD contains experimentally verified data, we compared the localizations to predictions for the wild type proteins. The analysis was made for localizations for which SP and/or WoLF PSORT make predictions.

The compartments with the largest numbers of proteins are plasma membrane, cytoplasm, nucleus, extracellular space, and mitochondria (see Additional file 1). Endoplasmic reticulum (ER) had the highest number of proteins as a secondary classification (see Additional file 2). Proteins were distributed unequally among the different compartments. Although disease-related proteins form a special group, they still reflect the overall properties of all proteins.

Table 1 indicates that results for TMHMM, TargetP predictions for mitochondrial proteins, PeroxiP and PTS1 have high accuracy while Golgipredictor and PredictNLS have only moderate performance. Precision values indicate that except for Golgipredictor and PeroxiP the predictors mainly detect the proteins well. However, when recall is considered, only TargetP and TMHMM are highly reliable. The overall parameter MCC values range from 0.056 to 0.58.

**Table 1:** Prediction results for the localization of wild type proteins with the individual predictors^a^

Compartment	tp	rp	tn	fn	Accuracy	Precision	Recall	MCC	Proteins located
TargetP (mitochodrial)	101	109	1254	52	0.894	0.481	0.660	0.506	210
TMHMM	377	144	853	142	0.811	0.724	0.726	0.581	521
Golgipredictor	23	178	227	20	0.558	0.114	0.535	0.056	201
PeroxiP	6	24	423	12	0.923	0.200	0.333	0.220	30
PTS1	3	1	446	15	0.966	0.750	0.167	0.343	4
PredictNLS	98	23	248	217	0.590	0.810	0.311	0.279	121
Summary					0.790^b^	0.513^b^	0.455^b^	0.331^b^	1087

^a^tp, the number of positive cases that were correctly predicted; tn, the number of negative cases correctly predicted; fp, the number of positive cases incorrectly predicted; fn, the number of negative cases incorrectly predicted.

^b^average value.

One reason for the poor behaviour of certain predictors is likely the fact that they are usually not used alone i.e. other programs are used to sort the data to localization routes before applying these tools. Overall, the methods obtained good precision at the cost of recall (false negatives). In summary, the individual methods can be applied with relatively high accuracy and precision to localization predictions. Methods, which predict the localization at the end of a complex pathway, are less reliable when applied directly to sequences.

### Reliability of the combined localization predictors

The SP predicted localization for 12 possible compartments and WoLF PSORT (animal version) predicted ten localizations. The results for the two approaches and comparison to experimental data for the wild type proteins are shown in Tables 2 and 3. Several parameters were calculated to describe the prediction performance. For the SP, altogether 60.9% (966/1586) of predictions were correct. Seventy proteins received two predictions in TargetP and thus two routes in SignalP. In these cases both predictions are included. No mitochondrial periplasmic space proteins were predicted and the false negative rate is very high for these cases. The precision and recall of Golgi, transmembrane is low as well as the values for peroxisomal localization. Accuracy and precision are usually clearly better than the recall values, which is in line with the results for individual predictors (Table 1). The results for gPM and mPM were combined to those for plasma membrane, since these localizations were predicted only for 3 and 6 proteins, respectively.

**Table 2:** Prediction results for the localization of wild type proteins with the Scandinavian protocol.

Compartment^a^	tp	fp	tn	fn	Accuracy	Precision	Recall	MCC	Proteins located
Mtm	10	29	1337	140	0.889	0.256	0.067	0.086	39
Mps	0	6	1363	147	0.899	0.000	0.000	-0.021	6
Mma	91	74	1293	58	0.913	0.552	0.611	0.532	165
Gtm	23	178	1240	75	0.833	0.114	0.235	0.079	201
PM	221	35	619	641	0.554	0.863	0.256	0.268	256
S	246	84	1093	93	0.883	0.745	0.726	0.661	330
ER	2	1	1330	183	0.879	0.667	0.011	0.074	3
N	98	23	1097	298	0.788	0.810	0.247	0.368	121
P	6	24	1462	24	0.968	0.200	0.200	0.184	30
C	269	166	852	229	0.739	0.618	0.540	0.392	435
Summary	966^b^	620^b^		1888^b^	0.835^c^	0.483^c^	0.289^c^	0.262^c^	1586^b^

Abbreviations for statistical parameters as in Table 1.

^a ^C, cytosol; Gtm, Golgi, transmembrane; Mma, mitochondrial matrix; Mps, mitochondrial, periplasmic space; Mtm, mitochondrial transmembrane; N, nuclear; P, peroxisomal; PM, plasma membrane; S, secreted

^b^total number

^c^average value.

**Table 3:** Prediction results for the localization of wild type proteins with WoLF PSORT.

	Mutant compartment								
Wild type compartment^a^	tp	fp	tn	fn	Accuracy	Precision	Recall	MCC	Proteins located
CK	2	38	1471	5	0.972	0.050	0.286	0.110	40
CK_PM	0	31	1485	31	0.960	0.000	0.000	-0.020	31
C	339	304	713	160	0.694	0.527	0.679	0.362	643
C_G	0	29	1450	37	0.956	0.000	0.000	-0.022	29
C_M	8	145	1328	35	0.881	0.052	0.186	0.048	153
C_N	102	365	963	86	0.703	0.218	0.543	0.191	467
C_P	3	77	1425	11	0.942	0.038	0.214	0.070	80
ER	85	253	1081	97	0.769	0.251	0.467	0.217	338
ER_G	4	70	1392	50	0.921	0.054	0.074	0.023	74
ER_M	2	92	1413	9	0.933	0.021	0.182	0.042	94
S	281	321	856	58	0.750	0.467	0.829	0.474	602
S_PM	4	148	1358	6	0.898	0.026	0.400	0.081	152
G	13	74	1344	85	0.895	0.149	0.133	0.085	87
L	33	202	1258	23	0.852	0.140	0.589	0.235	235
M	128	325	1038	25	0.769	0.283	0.837	0.394	453
M_N	0	33	1459	24	0.962	0.000	0.000	-0.019	33
M_P	1	112	1397	6	0.922	0.009	0.143	0.018	113
N	325	330	790	71	0.735	0.496	0.821	0.467	655
P	12	302	1184	18	0.789	0.038	0.400	0.068	314
PM	356	185	841	134	0.790	0.658	0.727	0.533	542
Summary	1696^b^	3398^b^		966^b^	0.854^c^	0.174^c^	0.375^c^	0.168^c^	5095^b^

Abbreviations for statistical parameters as in Table 1.

^a^C, cytosol; CK, cytoskeleton; G, Golgi compartment; M, mitochondrial; N, nuclear; P, peroxisomal; PM, plasma membrane; S, secreted

^b^total number. Underline sign indicates multiple predictions.

^c^average value

In the case of WoLF PSORT, 33.7% (1696/5095) gave correct predictions (Table 3). There are a number of dual predictions, eg for proteins, which shuttle between cytosol and nucleus. Results for these predictions were considered as correct only if the protein was found from both compartments. Values for accuracy ranged from 0.69 to 0.98 (average 0.854), whereas recall ranged from 0 to 0.84 (average 0.375). Peroxisomal proteins clearly had the lowest prediction accuracy. The results for WoLF PSORT do not allow a direct comparison with the SP, because WoLF PSORT considers combined predictions to be correct when one of the predictions is correct. Actually, just six classes had a substantial number of predicted proteins. The overall accuracy is almost identical for the two protocols whereas SP has clearly better precision and somewhat higher MCC score. The recall is slightly better for WoLF PSORT.

In conclusion, detailed analysis of the prediction performance indicates that the subcellular localization predictors still have much to improve. However, because the accuracy of individual predictions are rather high, these methods are indeed applicable to systematic analysis of mutations even though the precision, recall and MCC are clearly suboptimal. The more steps there are in the analysis the lower the expected accuracy (and other parameter values). Thus, if the analysis is based on five consecutive steps (as in SP) in which each step has 90% accuracy the final expected accuracy would be 59% (0.9^5^).

### Analysis of mutation effects

As the results above indicate, the subcellular localization of individual compartments of the investigated proteins can be predicted with rather high accuracy and also multipredictors provide useful data. The effect of mutations on protein localization was tested for all 22,416 missense mutations. In this analysis we looked for differences in predicted localization compared with that for wild type forms. Even if the prediction of the compartment was incorrect, a change in the predicted localization due to mutation might indicate the mutation mechanism and be useful for further studies. Similar effect has been useful also in some other bioinformatics predictions such as protein secondary structures.

The SP predicted that 203 mutations would alter protein localization. Results in Table 4 and in Additional file 3 show the distribution of mutations in the different subcompartments for the mutations and proteins in which the mutations appear, respectively. The numbers represent correctly predicted proteins and the total number of mutations for each category. The most common original compartments for proteins whose localization changed on account of the mutation were plasma membrane, Golgi transmembrane, nucleus, and cytoplasm. Most common among the mutant sublocalizations were plasma membrane, Golgi transmembrane, and nucleus. The single most common predicted mutation type was from plasma membrane (for wild type localization) to Golgi transmembrane–altogether 47 cases, 17 of which had the correct prediction for the wild type form. Although the number of correct wild type predictions was not directly related to the mutation predictions, the numbers varied widely–as an extreme case C to N prediction, with 19 of 20 having the correct wild type prediction for 11 proteins out of 12. The range of changes to localizations of mutations varied from one to five, the highest being for Gtm proteins. Similarly, the predicted range of mislocalizations from one subcompartment to others varied from one to six, with cytoplasmic proteins being redirected to six different compartments when mutated.

**Table 4:** Changes in SP localization prediction due to mutations.

	Mutant compartment															
Wild type compartment^a^	Gtm	Mtm/Gtm	Mma/C	Mma	Mma/PM	Mps/S	C	Mma/P	N	PM	Mtm	S	Mtm/PM	Mma/N	P	Total
Mtm/Gtm	0/3				1/1											1/4
Mtm/PM	0/6	0/9			1/1											1/16
PM	17/47						0/1						4/5			21/53
S	0/4									7/8						7/12
C	0/1		8/8						19/20	1/1		1/1			0/2	29/33
Mtm		0/3		1/1												1/4
Mma/Gtm		0/2														0/2
Gtm		0/5								17/21	0/1	2/4				19/31
Mtm/C			0/1	1/4												1/5
Mps/C			1/1													1/1
Mma			6/6			1/1	2/2	1/1								10/10
Mma/S			2/2	1/1		1/1										4/4
Mma/C							4/4									4/4
Mps						2/2										2/2
Mma/N							1/1		1/1							2/2
P									1/1							1/1
N										0/18				1/1		1/19

Total	17/61	0/19	17/18	3/6	2/2	4/4	7/8	1/1	21/22	25/48	0/1	3/5	4/5	1/1	0/2	105/203

The numbers separated by the slash sign are for how many proteins the wild type localization has been correctly predicted, and the number of analyzed mutations, respectively.

^a^C, cytosol; Gtm, Golgi, transmembrane; Mma, mitochondrial matrix; Mps, mitochondrial, periplasmic space; Mtm, mitochondrial transmembrane; N, nuclear; P, peroxisomal; PM, plasma membrane; S, secreted. Slash sign indicates alternative predicted localizations.

Results for the mutations and proteins analyzed by WoLF PSORT are shown in Table 5 and Additional file 4, respectively. To avoid excessive partitioning of the results to very small groups, only the results for the highest prediction score are indicated. About 50% of the wild type proteins had the correct localization. Altogether, WoLF PSORT found 183 cases with predicted alteration caused by mutation. The highest number of mutation-based rerouting to other compartments was for proteins whose wild type form was predicted to localize to the cytoplasm. Extracellular, cytoplasm, plasma membrane, nuclear and mitochondria are the most common localizations for mutant proteins. In comparison to SP, WoLF PSORT had somewhat lower numbers in target compartments. The changes with the largest number of mutations were CN to C, and N to C, which are related predictions. WoLF PSORT may suffer from using BLAST as part of its algorithm. In the case of SP, the search for homologues was not implemented, however, that was not possible to do for WoLF PSORT.

**Table 5:** Changes in WoLF PSORT localization prediction due to mutations.

	Mutant compartment																	
Wild type compartment^a^	PM	S	S_PM	C	C_N	C_M	N	N/C_N	N/C/ C_N	ER	ER_ M	M	M_N	M_N/ C_M	P	L	CK	Total
P		2/2	0/1	1/6			2/2			0/1		0/6			1/1			6/19
S	0/1				0/4		0/1			4/5	1/1	4/9				0/2		9/23
S/S_PM										0/1								0/1
C	0/6	2/3		3/9		3/3	5/6	0/1				3/6					2/2	18/36
C/C_N							1/1											1/1
C_N		0/2		18/19			4/8					2/2	2/2	3/3				29/36
C_M				1/1														1/1
N	0/1	0/10		6/10	7/7							2/4						15/32
G/ER_G																0/1		0/1
ER	0/11	0/1		1/1								0/1			2/2			3/16
M	0/2	3/3		6/6			0/2	0/2										9/15
ER_M				1/1														1/1
M/P/M_P									0/1									0/1

Total	0/21	7/21	0/1	37/53	7/11	3/3	12/20	0/3	0/1	4/7	1/1	11/28	2/2	3/3	3/3	0/3	2/2	92/183

The numbers separated by the slash sign are for how many proteins the wild type localization have been correctly predicted, and the number of analyzed mutations, respectively.

^a^C, cytosol; CK, cytoskeleton; G, Golgi compartment; M, mitochondrial; N, nuclear; P, peroxisomal; PM, plasma membrane; S, secreted. Slash sign indicates alternative localization predictions and underline sign multiple predicted localizations.

The results for the identified changes in protein localization due to missense mutations are shown in Additional file 5 and Additional file 6. The two prediction approaches, SP and WoLF PSORT, agreed on 17267 (77%) of the total 22,416 mutations when all predictions of WoLF PSORT were taken into account. Of the two approaches, 203 and 183 mutations were predicted to alter the target compartments of mutant proteins, affecting 105 and 92 proteins, respectively. 18 of these proteins were common for the two methods, and in these proteins the protocols agreed on 12 mutations to affect proteins localization. The two methods predicted the same compartment mislocalization in seven cases. This indicates that neither of the methods was able to detect all putative localization mutations. Similar result calling for use of several tools was apparent when splice site prediction tools were tested for mutation analysis [18].

We can estimate the number of expected mutations in localization sites. Our data set contains 1,516 proteins, which consist of 1,054,823 amino acid residues, and which have 2373 localizations based on HPRD. The length of the targeting peptides varies from a few residues to close to 30. If we use an average value of eight residues for the targeting peptide, we should see 403 (2373*8/1054823*22416) mutations in localization signals. This number is almost exactly what was observed.

**Figure 1:**
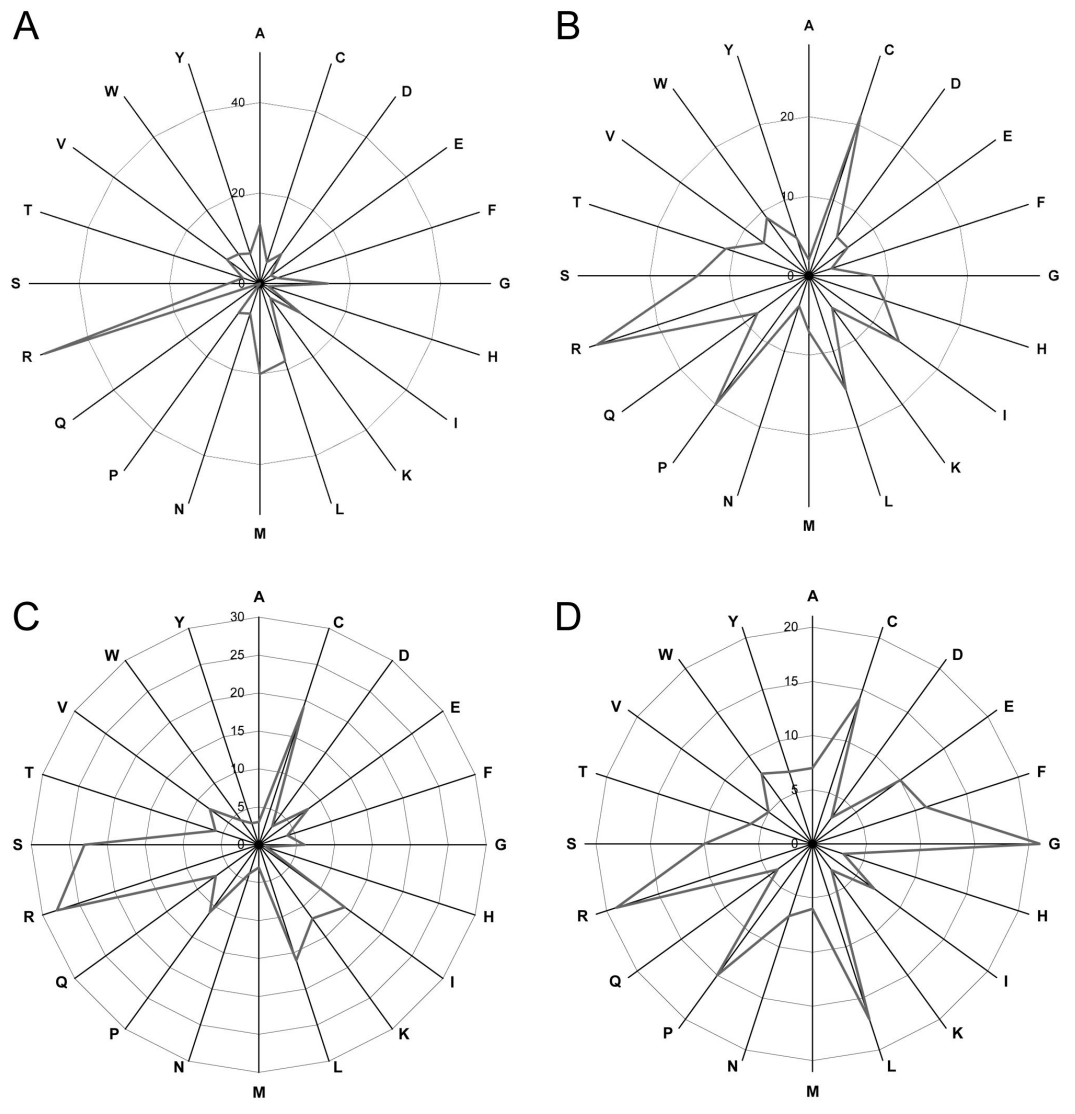
**Amino acid distribution for the two prediction schemes**. Scandinavian protocol (A, B) and WoLF PSORT (C, D) predictions of (A, C) amino acids in wild type proteins that are predicted to be mutated in localization mutants, and (B, D) mutant amino acids in localization mutants.

**Figure 2:**
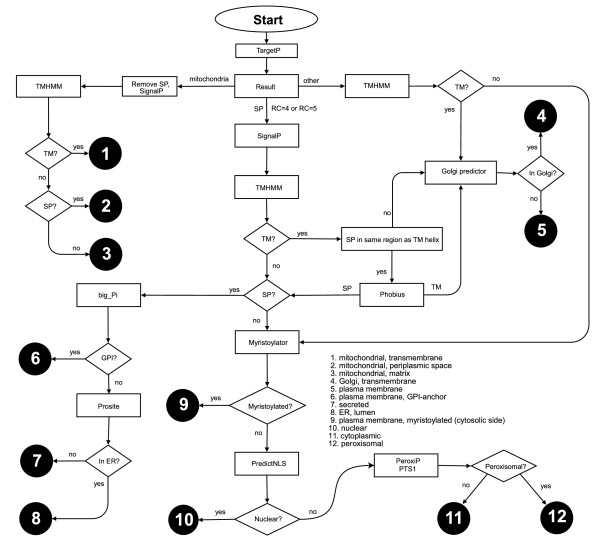
**Schematic illustration of the analysis of protein localization with the Scandinavian protocol**. The predicted compartments are indicated with corresponding numbers in black circles. The localizations are listed in the middle of the figure. RC, reliability coefficient; SP, signal peptide; TM, transmembrane region.

The distribution of the amino acid changes in the predicted localization alterations is shown in Fig. 1. The amino acid distributions for mutations were compared with information for all human proteins taken from Codon Usage Tabulated from GenBank (CUTG) [19]. The distribution of all the mutations was significantly biased compared to random distribution in all amino acid types except for D and H (see Additional file 7). The results are in line with previous mutation distribution studies for numerous proteins and secondary structural elements within them [20-22], including mutations in the protein kinase family [23] and in immunodeficiencies [12]. These studies indicated highly skewed distribution for disease mutations, which varies also between secondary structural elements. Data for the SP indicated that mutations are most common in R (Fig. 2 and Additional file 8). Arginine is coded by six synonymous codons, four of which contain a CpG dinucleotide, a well known mutational hot spot [24]. Also G, L and M are frequently mutated. The most common mutant residues were R, C, and P, of which arginine is the most common. Arginine was usually replaced by C (14 of 50 cases), making this the single most frequent mutation type. Eight of 9 mutations to W were from R, and 7 of 8 mutations in Q were from arginine. Arginine was mutated altogether to 10 other residues, i.e., all except two (K and M) of the possible substitutions with single nucleotide changes. Arginine was also the most common resulting residue from mutations in other codons, and it was the residue type with the highest number of original residues, 9. Somewhat surprisingly, no localization mutations were identified in Q, which however occurred 8 times as a mutant residue.

Of note, only two mutations to A and three mutations to F were predicted to be disease-related. H, Q and E were the least frequently mutated residues. These results follow somewhat the general amino acid distribution with prominent exceptions like arginine.

WoLF PSORT results show some differences from SP, which may have originated from the prediction algorithm. R, G, and L were the most commonly mutated residues. However, arginine did not show the clear overprediction as in the SP data. D, H and K were the least mutated residues (Fig. 2 and Additional file 9). Mutations to G and R both appeared in seven original residue types, whereas S was mutated from eight original residues. These were also the residues that had the highest number of mutant residue types. Only one change to H, two to N, D or I were predicted to be related to diseases.

### Comparison to known mislocalization mutations

Our results predicted localization changes that underlie many different types of diseases, including those involving signal transduction, metabolism, immunodeficiencies, eye diseases, developmental disorders and cancers (see Additional file 5 and Additional file 6). Some disease-related mutations, which have been confirmed to affect protein localization, have been described. These cases are usually sporadic in the literature. Because no database is available for such mutations, we performed a literature search and identified a number of cases.

Mutations in SHOX, homeobox-containing gene, cause idiopathic short stature, Leri-Well dyschondrosteosis and Langer mesomelic dysplasia. The substitution R173C prevents the transport of the *SHOX*-encoded protein to the nucleus and its subsequent function as a transcription activator [25]. Both the SP and WoLF PSORT correctly predicted the mislocalization and the effect of the mutation.

AIRE, autoimmune regulator, is a nuclear protein and transcriptional regulator. Wild type AIRE appears both in nuclear dots, as evenly distributed in the nucleus, and in the cytoplasm. Several mutations have been shown to affect the distribution of AIRE between compartments [26-28]. Mutations R14L, T16M, A21V and Y85C were correctly predicted to affect protein localization by the SP and L28P and L29P by WoLF PSORT predictor. However, the predicted changes were not accurate, because the SP has a change from cytoplasmic and mitochondrial matrix to cytoplasm and WoLF PSORT from secreted to mitochondrial.

Similar results were obtained for *BSND *mutations. Barttin, encoded by *BSND*, is involved in Bartter syndrome, a renal tubular salt-wasting disease. Barttin localizes to the plasma membrane, whereas mutant forms are retained in the ER [29]. R8L was predicted by SP to change the localization from Golgi transmembrane to plasma membrane. A milder form, G10S, which appears in both the ER and the plasma membrane, was not predicted to affect localization. We also consider this kind of prediction useful because a localization change is forecast due to the mutation. Thus, the predictions can give a hint of the possible mechanism, even though the final validation must be obtained experimentally.

We did predictions for cases, which according to liture affect the localization in *ATP7B *mutations in Wilson disease [30], *ABCA1 *mutations in Scott syndrome [31], *RPS19 *mutations in Diamond-Blackfan anemia [32], *ABCA1 *mutations in Tangier disease [33], and laminin A/C mutations in heritable dilated cardiomyopathy [34]. However, the predictions agreed with the experimental data only for the *FXYD2 *mutation in hereditary primary hypomagnesia [35]. These results show the poor recall of the methods.

Several reasons account for failure of the predictions to detect all the localization changes. As noted above, the predictions are characterized by high accuracy and low recall. Even the experimental information can sometimes be misleading, because the localization effect can be secondary and may not have been investigated in detail. In the androgen receptor C169Y missense mutation, mutant receptor aggregation causes a change in localization [36]. The wild type protein is in nucleoplasm whereas the mutant forms aggregate in both nucleus and cytoplasm. In nine mutations in nonmuscle myosin heavy chain A (MYH9), the mutant proteins aggregate, causing several disorders characterized by giant platelets, thrombocytopenia, and Döhle body-like cytoplasmic inclusions in granulocytes [37].

Sometimes localization-changing mutations appear outside the targeting signals. Forkhead box (FOX) P2 involved in a speech/language disorder has two separate nuclear localization signals. Mutant protein R553H is mainly targeted to the cytoplasm instead of the nucleus [38]. The mutation appears in the region between but not within the two nuclear localization signal sequences. Neutral evolution can generate novel targeting signals. Putative peroxisomal targeting signals were identified from a number of non-peroxisomal proteins and were shown to have a potential to be activated if the original target signal is changed or not accessible [39]. A mutation in the pleckstrin homology domain of AKT1 kinase leads to cancer because of pathological localization to the plasma membrane [40]. AKT1 is normally translocated from nucleus to the plasma membrane in response to growth factor stimulation. A mutant form of E17K, which has increased phosphorylation, is located at the plasma membrane in response to growth factor stimulation.

Cell type-specific alternative splicing can alter the localization of proteins, including myotonic dystrophy protein kinase (DMPK) [41]. Still another mechanism affects the Menkes disease copper ATPase, in which the mutation G1019D interferes with protein folding [42]. Similar effects have been seen for certain breast cancer 1 (BRCA1) mutations [43]. In tafazzin, mutations disrupt the membrane association region [44].

The localization of tyrosine phosphatase SHP-1 is regulated by phosphorylation [45] and alternative start sites [46,47]. The predictions for mutations in any of these proteins indicated no changes to localization.

## Conclusion

Applicability of protein localization prediction methods were tested in detecting changes in localization due to point mutations. Altogether 374 mutations were predicted by at least one method to affect protein localization. Because disease mutations are unequally distributed throughout protein sequences, having a higher occurrence in structurally/functionally important sites, we can expect the number of localization mutations to be higher than calculated. The expected number is 403 mutations. Localization mutations are rare events, but they should be taken into account when predicting consequences of mutations. A service for SP predictions will be released in the near future as part of the Pathogenic-Or-Not -Pipeline (PON-P, ).

## Methods

### Mutation, localization and sequence data

Missense mutations were obtained from the HGMD [48] (downloaded 17.3.2007) and IDbases [12]. The dataset was filtered to include only genes for which cDNA sequence was available. The experimental localization(s) of each identified protein was collected from the Human Protein Reference Database (HPRD, ) [3] (4.5.2007). We excluded proteins for which the experimental localization was unknown. After these filtering steps, 1,516 proteins remained, which contained altogether 22,416 missense mutations (on average 14.8 per protein). Altogether, we identified 2,373 localizations, indicating that the average per-protein localization was ~1.6 for the 1,516 proteins we investigated. The proteins had 34 primary localizations (Table 1) and altogether 56 localizations (Additional file 2).

We identified both the first (most common) and all localizations for each protein. The wild type protein sequences were translated from cDNA sequences obtained from HGMD. The disease-related mutations were introduced into the protein sequences one by one and analyzed individually. Programs and scripts for the analysis were written in Java or Perl languages.

### Localization prediction methods

First, predictions were made separately for certain localizations and then by two strategies for combined predictions. Groups in Stockholm, Sweden, and Lyngby, Denmark, whose long-term efforts have resulted in methods for numerous tasks in subcellular localization prediction, recently published a protocol to combine different predictions developed by them and others into a comprehensive prediction scheme [49]. This Scandinavian protocol (SP) is rather complicated and requires the use of numerous separate prediction tools. To facilitate the analysis, we developed a program that automatically runs all the predictions, parses the results, and provides the outcome of the prediction. The flow chart for the analysis steps and programs is in Fig 2. As a modification, nuclear localization signals were predicted only with the PredictNLS program. Because we analyzed human proteins, it was not necessary to investigate chloroplast localization or prokaryotic predictions. Some of the programs use database searches to identify homologues to strengthen the predictions. We had to omit this step because the wild type sequences in the databases are identical to the mutant sequences, apart from the single missense mutation. Because sequence conservation is indicative of protein colocalization [50,51], database searches would have selected the wild type sequence for prediction and thereby hampered the analysis of mutants. Also the step for β-barrel prediction was omitted.

The programs TargetP [52], SignalP [53] and TMHMM [54,55] were downloaded from  and were run locally, whereas programs Big-PI [56-59], NMT , PeroxiP [60], PredictNLS [11], PTS1 [61,62], Golgipredictor [63], Phobius [64] and Prosite [65] were run over the Internet. Altogether, this procedure could predict 12 different localizations (Fig 1).

In the SP protocol, first the TargetP assigns whether the proteins go to mitochondia or secretory pathway or not. The mitochondrial proteins are classified further to transmembrane, periplasmic space or matrix based on the analysis of transmembrane and signal peptide sequences. Transmembrane proteins are predicted via two routes and are then classified to those ending in Golgi transmembrane or plasma membrane. Signal peptide(s) containing proteins are classified to different compartments whether they contain transmembrane region(s), signal peptide, are myristoylated, have GPI anchors or are predicted to endoplasmic reticulum.

The other method we applied, WoLF PSORT, is an integrated program that makes predictions for 10 subcellular compartments [66]. WoLF PSORT was run locally with default parameters. WoLF PSORT program was downloaded from  and run locally.

We ran each prediction strategy for both wild type and mutated sequences and determined whether the mutation(s) changed the localization prediction. Both protocols may predict multiple localizations for a protein–for example nucleus and cytosol for a protein that is transported between nucleus and cytosol. Thus, all highest-score predictions provided by the programs were taken into account. In SP, if TargetP had problems to resolve the localization for a protein predicted to mitochondria with poor reliability coefficient (RC) (value 4 or 5) then the protein was predicted also with SignalP and it gets two alternative localizations (Fig 1).

The two methods predict proteins to following compartments. The SP predicted localization for 12 possible compartments, which include the mitochondrial membrane (transmembrane) (Mtm), mitochondrial periplasmic space (Mps), mitochondrial matrix (Mma), Golgi, transmembrane (Gtm), plasma membrane (PM), secreted (S), ER lumen (ER), nucleus (N), peroxisome (P), cytoplasmic C), plasma membrane, GPI anchor (gPM), and plasma membrane, myristoylated (mPM). WoLF PSORT (animal version) predicted ten localizations: cytosol (C), cytoskeleton (CK), ER, extracellular (S), Golgi apparatus (G), lysosome (L), mitochondria (M), nucleus (N), peroxisome (P) and plasma membrane (PM). All these were present in the dataset.

The quality of the predictions was measured by four parameters: accuracy, recall, precision, and the Matthew's correlation coefficient (MCC) as follows:



where tp is the number of positive cases that were correctly predicted, tn is the number of negative cases correctly predicted, fp is the number of positive cases incorrectly predicted, and the fn is the number of negative cases incorrectly predicted.

## Abbreviations

C: cytoplasmic; CK: cytoskeleton; ER: ER lumen; fn: false negative. false positive; G: Golgi apparatus; gPM: plasma membrane, GPI anchor; Gtm: Golgi, transmembrane; HGMD: Human Gene Mutation Database; HPRD: Human Protein Reference Database; L: lysosome; M: mitochondria; Mma: mitochondrial matrix; mPM: plasma membrane, myristoylated; Mps: mitochondrial periplasmic space; Mtm: mitochondrial membrane, transmembrane; N: nucleus; OMIM: Online Mendelian Inheritance in Man; P: peroxisome; PM: plasma membrane; RC: reliability coefficient; S: secreted; SP: Scandinavian Protocol; tn: true negative; tp: true positive.

## Authors' contributions

KL collected data, performed the statistical analysis and drafted the manuscript. MV conceived of the study, designed the study, analyzed the data and drafted the manuscript. All authors read and approved the final manuscript.

## Supplementary Material

Additional File 1 **The primary localization of the proteins according to HPRD**. Number of proteins localizing according to HPRD (first localization only). Click here for file

Additional File 2 **All localizations of the proteins according to HPRD**. Information about all the localizations for the studied proteins. Click here for file

Additional File 3 **Changes in SP localization prediction due to mutations (data for affected proteins)**. Information for mutations related to diseases according to Scandinavian protocol. Click here for file

Additional File 4 **Changes in WoLF PSORT localization prediction due to mutations (data for affected proteins)**. Information for mutations related to diseases according to WoLF PSORT. Click here for file

Additional File 5 **Mutations predicted by SP to alter protein localization**. List of disease-causing mutations predicted to be related to protein localization by SP. Click here for file

Additional File 6 **Mutations predicted by WolF PSORT to alter protein localization**. List of disease-causing mutations predicted to be related to protein localization by WoLF PSORT. Click here for file

Additional File 7 **Comparison of observed and expected numbers of mutations in the dataset**. Statistical analysis of numbers of mutations. Click here for file

Additional File 8 **Amino acid distribution of localization mutations predicted with SP**. Statistics of amino acid changes in the disease-causing mutations predicted to change localization by SP. Click here for file

Additional File 9 **Amino acid distribution of localization mutations predicted with WolF PSORT**. Statistics of amino acid changes in the disease-causing mutations predicted to change localization by WoLF PSORT. Click here for file
