# Supplementary material for: Prediction of disease-related mutations affecting protein localization
Source: BMC Genomics. 2009 Mar 23;10:122. doi: 10.1186/1471-2164-10-122 (PMC2680896; doi:10.1186/1471-2164-10-122)
Supplement: Additional File 1 — The primary localization of the proteins according to HPRD. Number of proteins localizing according to HPRD (first localization only). [file 1471-2164-10-122-S1.doc]

Additional file 1 - The primary localization of the proteins according to HPRD

Compartment Number of proteins

Plasma membrane 391

Cytoplasm 275

Nucleus 250

Extracellular 234

Mitochondrion 123

Endoplasmic reticulum 88

Lysosome 35

Golgi apparatus 27

Integral to membrane 19

Peroxisome 17

Nucleolus 8

Sarcoplasmic reticulum 8

Endosome 6

Cytoplasmic vesicle 5

Centrosome 3

Microsome 2

Endoplasmic reticulum membrane 2

Nucleoplasm 2

Nuclear membrane 2

Perinuclear region 2

Cytoskeleton 2

Cell junction 2

Mitochondrial membrane 2

Tubulin 1

Cell surface 1

Membrane fraction 1

Secretory granule 1

Dendrite 1

Secreted 1

Extracellular matrix 1

Cytosol 1

Peroxisomal membrane 1

Basolateral membrane 1

Mitochondrial intermembrane space 1

Total 1516

Number of proteins localizing according to HPRD (first localization only).
