# Supplementary material for: Prediction of disease-related mutations affecting protein localization
Source: BMC Genomics. 2009 Mar 23;10:122. doi: 10.1186/1471-2164-10-122 (PMC2680896; doi:10.1186/1471-2164-10-122)
Supplement: Additional File 2 — All localizations of the proteins according to HPRD. Information about all the localizations for the studied proteins. [file 1471-2164-10-122-S2.doc]

## Additional file 2 - All localizations of the proteins according to HPRD

Compartment Number of proteins

Plasma membrane 481

Cytoplasm 477

Nucleus 368

Extracellular 326

Endoplasmic reticulum 170

Mitochondrion 147

Golgi apparatus 97

Lysosome 56

Nucleolus 42

Peroxisome 28

Endosome 28

Integral to membrane 27

Cytoplasmic vesicle 13

Centrosome 11

Sarcoplasmic reticulum 10

Secretory granule 9

Microsome 7

Perinuclear region 6

Cytoskeleton 6

Nuclear membrane 4

Cytosol 4

Desmosome 4

Endoplasmic reticulum membrane 3

Membrane fraction 3

Extracellular matrix 3

Mitochondrial membrane 3

Cell surface 2

Nucleoplasm 2

Cell junction 2

Secreted 2

Mitochondrial matrix 2

Ribosome 2

Early endosome 2

Synapse 2

Nuclear matrix 2

Kinetochore 2

Tubulin 1

Dendrite 1

Peroxisomal membrane 1

Basolateral membrane 1

Mitochondrial intermembrane space 1

Late endosome 1

Cilium 1

Cell projection 1

Microtubule 1

Axon 1

Perinuclear vesicle 1

Vesicle 1

Secretory vesicle 1

Peroxisomal matrix 1

Soluble fraction 1

Actin cytoskeleton 1

Clathrin-coated vesicle 1

Golgi membrane 1

Axoneme 1

Centriole 1

Total 2373
