# Supplementary material for: Prediction of disease-related mutations affecting protein localization
Source: BMC Genomics. 2009 Mar 23;10:122. doi: 10.1186/1471-2164-10-122 (PMC2680896; doi:10.1186/1471-2164-10-122)
Supplement: Additional File 3 — Changes in SP localization prediction due to mutations (data for affected proteins). Information for mutations related to diseases according to Scandinavian protocol. [file 1471-2164-10-122-S3.doc]

## Additional file 3 - Changes in SP localization prediction due to mutations (data for affected proteins).

Mutant compartment

| Wild type compartment | Gtm | Mtm/ Gtm | Mma/C | Mma | Mma/ PM | Mps/S | C | Mma/P | N | PM | Mtm | S | Mtm/ PM | Mma/N | P | Total |
| --- | --- | --- | --- | --- | --- | --- | --- | --- | --- | --- | --- | --- | --- | --- | --- | --- |
| Mtm/Gtm | 0/1 |  |  |  | 1/1 |  |  |  |  |  |  |  |  |  |  | 1/2 |
| Mtm/PM | 0/1 | 0/4 |  |  | 1/1 |  |  |  |  |  |  |  |  |  |  | 1/6 |
| PM | 2/10 |  |  |  |  |  | 0/1 |  |  |  |  |  | 2/3 |  |  | 4/14 |
| S | 0/1 |  |  |  |  |  |  |  |  | 4/5 |  |  |  |  |  | 4/6 |
| C | 0/1 |  | 3/3 |  |  |  |  |  | 11/12 | 1/1 |  | 1/1 |  |  | 0/1 | 16/19 |
| Mtm |  | 0/1 |  | 1/1 |  |  |  |  |  |  |  |  |  |  |  | 1/2 |
| Mm/Gtm |  | 0/1 |  |  |  |  |  |  |  |  |  |  |  |  |  | 0/1 |
| Gtm |  | 0/1 |  |  |  |  |  |  |  | 10/14 | 0/1 | 2/4 |  |  |  | 12/20 |
| Mtm/C |  |  | 0/1 | 1/3 |  |  |  |  |  |  |  |  |  |  |  | 1/4 |
| Mps/C |  |  | 1/1 |  |  |  |  |  |  |  |  |  |  |  |  | 1/1 |
| Mma |  |  | 3/3 |  |  | 1/1 | 1/1 | 1/1 |  |  |  |  |  |  |  | 6/6 |
| Mma/S |  |  | 1/1 | 1/1 |  | 1/1 |  |  |  |  |  |  |  |  |  | 3/3 |
| Mma/C |  |  |  |  |  |  | 3/3 |  |  |  |  |  |  |  |  | 3/3 |
| Mps/C |  |  |  |  |  | 1/1 |  |  |  |  |  |  |  |  |  | 1/1 |
| Mma/N |  |  |  |  |  |  | 1/1 |  | 1/1 |  |  |  |  |  |  | 2/2 |
| P |  |  |  |  |  |  |  |  | 1/1 |  |  |  |  |  |  | 1/1 |
| N |  |  |  |  |  |  |  |  |  | 0/1 |  |  |  | 1/1 |  | 1/2 |
| Total | 2/14 | 0/7 | 8/9 | 3/5 | 2/2 | 3/3 | 5/6 | 1/1 | 13/14 | 15/21 | 0/1 | 3/5 | 2/3 | 1/1 | 0/1 | 58/93 |

The numbers separated by the slash sign are for how many mutation containing proteins the wild type localization have been correctly predicted, and the number of proteins with mutations, respectively.

C, cytosol; Gtm, Golgi, transmembrane; Mma, mitochondrial matrix; Mps, mitochondrial, periplasmic space; Mtm, mitochondrial transmembrane; N, nuclear; P, peroxisomal; PM, plasma membrane; S, secreted. Slash sign indicates alternative localization predictions.
