# Supplementary material for: Prediction of disease-related mutations affecting protein localization
Source: BMC Genomics. 2009 Mar 23;10:122. doi: 10.1186/1471-2164-10-122 (PMC2680896; doi:10.1186/1471-2164-10-122)
Supplement: Additional File 4 — Changes in WoLF PSORT localization prediction due to mutations (data for affected proteins). Information for mutations related to diseases according to WoLF PSORT. [file 1471-2164-10-122-S4.doc]

## Additional file 4 - Changes in WoLF PSORT localization prediction due to mutations (data for affected proteins)

Mutant compartment

| Wild type compartment | PM | S | S_PM | C | C_N | C_M | N | N/ C_N | N/C/ C_N | ER | ER_M | M | M_N | M_N/ C_M | P | L | CK | Total |
| --- | --- | --- | --- | --- | --- | --- | --- | --- | --- | --- | --- | --- | --- | --- | --- | --- | --- | --- |
| PM |  | 2/2 | 0/1 | 1/3 |  |  | 2/2 |  |  | 0/1 |  | 0/3 |  |  | 1/1 |  |  | 6/13 |
| S | 0/1 |  |  |  | 0/1 |  | 0/1 |  |  | 2/3 | 1/1 | 3/7 |  |  |  | 0/2 |  | 6/16 |
| S/S_PM |  |  |  |  |  |  |  |  |  | 0/1 |  |  |  |  |  |  |  | 0/1 |
| C | 0/4 | 1/2 |  | 3/5 |  | 2/2 | 5/6 | 0/1 |  |  |  | 3/4 |  |  |  |  | 2/2 | 16/26 |
| C/C_N |  |  |  |  |  |  | 1/1 |  |  |  |  |  |  |  |  |  |  | 1/1 |
| C_N |  | 0/1 |  | 2/3 |  |  | 4/6 |  |  |  |  | 1/1 | 1/1 | 1/1 |  |  |  | 9/13 |
| C_M |  |  |  | 1/1 |  |  |  |  |  |  |  |  |  |  |  |  |  | 1/1 |
| N | 0/1 | 0/4 |  | 3/6 | 3/3 |  |  |  |  |  |  | 2/3 |  |  |  |  |  | 8/17 |
| G/ER_G |  |  |  |  |  |  |  |  |  |  |  |  |  |  |  | 0/1 |  | 0/1 |
| ER | 0/2 | 0/1 |  | 1/1 |  |  |  |  |  |  |  | 0/1 |  |  | 1/1 |  |  | 2/6 |
| M | 0/1 | 1/1 |  | 2/2 |  |  | 0/1 | 0/1 |  |  |  |  |  |  |  |  |  | 3/6 |
| ER_M |  |  |  | 1/1 |  |  |  |  |  |  |  |  |  |  |  |  |  | 1/1 |
| M/P/M_P |  |  |  |  |  |  |  |  | 0/1 |  |  |  |  |  |  |  |  | 0/1 |
| Total | 0/9 | 4/11 | 0/1 | 14/22 | 3/4 | 2/2 | 12/17 | 0/2 | 0/1 | 2/5 | 1/1 | 9/19 | 1/1 | 1/1 | 2/2 | 0/3 | 2/2 | 53/103 |

The numbers separated by the slash sign are for how many mutation containing proteins the wild type localization have been correctly predicted, and the number of proteins with mutations, respectively.

C, cytosol; CK, cytoskeleton; G, Golgi compartment; M, mitochondrial; N, nuclear; P, peroxisomal; PM, plasma membrane; S, secreted. Underline sign indicates multiple predictions and slash sign alternative predictions.
