# Supplementary material for: Prediction of disease-related mutations affecting protein localization
Source: BMC Genomics. 2009 Mar 23;10:122. doi: 10.1186/1471-2164-10-122 (PMC2680896; doi:10.1186/1471-2164-10-122)
Supplement: Additional File 5 — Mutations predicted by SP to alter protein localization. List of disease-causing mutations predicted to be related to protein localization by SP. [file 1471-2164-10-122-S5.doc]

## Additional file 5 - Mutations predicted by SP to alter protein localization

| Gene | Mutation | Wildtype localization | Mutant localization | Disease |
| --- | --- | --- | --- | --- |
| *ABCA4* | L1388P | PM | Gtm | Stargardt disease |
| *ABCA4* | L1390P | PM | Gtm | Cone-rod dystrophy |
| *ABCD1* | R104C | Mma/C | Mtm/C | Adrenoleukodystrophy |
| *ABO* | V36F | PM | Gtm | Blood group variation |
| *ACVRL1* | A128D | PM | S | Haemorrhagic telangiectasia 2 |
| *ADRB2* | R16G | Mtm/PM | PM | Asthma, nocturnal, association with |
| *AGTR2* | I53F | PM | Gtm | Mental retardation, X-linked |
| *AIRE* | A21V | Mma/C | C | APECED^a^ |
| *AIRE* | R15C | Mma/C | C | APECED |
| *AIRE* | R15L | Mma/C | C | APECED |
| *AIRE* | T16M | Mma/C | C | APECED |
| *AIRE* | Y85C | Mma/C | C | APECED |
| *AIRE* | Y90C | Mma/C | C | APECED |
| *AIRE* | V80L | Mma/C | C | APECED |
| *ATP2C1* | G789R | Gtm | PM | Hailey-Hailey disease |
| *AVPR2* | A285P | Mtm/Gtm | Mtm/PM | Diabetes insipidus, nephrogenic |
| *AVPR2* | A294P | Mtm/Gtm | Mtm/PM | Diabetes insipidus, nephrogenic |
| *AVPR2* | L282P | Mtm/Gtm | Mtm/PM | Diabetes insipidus, nephrogenic |
| *AVPR2* | L289P | Mtm/Gtm | Mtm/PM | Diabetes insipidus, nephrogenic |
| *AVPR2* | L292P | Mtm/Gtm | Mtm/PM | Diabetes insipidus, nephrogenic |
| *BBS2* | N70S | Mma/C | Mma | Bardet-Biedl syndrome |
| *BBS2* | V75G | Mma/C | Mma | Bardet-Biedl syndrome |
| *BCHE* | T24M | S | Gtm | Butyrylcholinesterase deficiency |
| *BRCA1* | R507I | N | C | Breast cancer |
| *BSND* | R8L | Gtm | PM | Bartter syndrome with sensorineural deafness |
| *BTD* | A82D | Mps/S | Mps | Biotinidase deficiency |
| *BTD* | D104Y | Mps/S | Mma/S | Biotinidase deficiency |
| *BTD* | G45R | Mps/S | Mma | Biotinidase deficiency |
| *BTD* | L71P | Mps/S | Mps | Biotinidase deficiency |
| *BTK* | K12R | C | Mma/N | Agammaglobulinaemia |
| *BTK* | K27R | C | Mma/C | Agammaglobulinaemia |
| *CDA* | K27Q | N | C | ara-C resistance, association with |
| *CFTR* | R31C | Mtm/Gtm | Gtm | Pancreatitis, idiopathic |
| *CFTR* | R3W | Mtm/Gtm | Gtm | Cystic fibrosis |
| *CFTR* | D993Y | Mtm/Gtm | Mtm/PM | Cystic fibrosis |
| *CLDN16* | R149L | Mtm/Gtm | Mtm/PM | Hypomagnesaemia, renal |
| *CPT1A* | R123C | Gtm | PM | Carnitine palmitoyltransferase 1 deficiency |
| *CX3CR1* | V249I | PM | Gtm | HIV infection, susceptibility to, association |
| *CYP11B1* | P42S | Mma | Mma/S | Steroid-11 beta-hydroxylase deficiency |
| *EDA* | C60R | Gtm | C | Ectodermal dysplasia |
| *EDA* | L55R | Gtm | C | Ectodermal dysplasia |
| *EDA* | M1L | Gtm | C | Ectodermal dysplasia |
| *EDA* | Y61H | Gtm | C | Ectodermal dysplasia |
| *ELN* | A707D | PM | S | Supravalvular aortic stenosis |
| *ENPP1* | L39P | PM | C | Myelopathy (OPLL)^b^ |
| *ETFDH* | P456L | C | PM | Etaricacidaemia 2c |
| *FGFR2* | G384R | PM | S | Craniosynostosis |
| *FGFR3* | A391E | PM | S | Crouzon syndrome |
| *FGFR3* | G380R | PM | S | Achondroplasia |
| *FGFR3* | G380R | PM | S | Achondroplasia |
| *FKRP* | M1V | S | Gtm | Muscular dystrophy, limb girdle |
| *FXYD2* | G41R | PM | Gtm | Hypomagnesaemia, renal |
| *GATA1* | R216Q | N | C | Tombocytopaenia 1 |
| *GATA1* | D218G | N | C | Tombocytopaenia 1 |
| *GATA1* | D218Y | N | C | Tombocytopaenia 1 |
| *GCK* | R36W | N | C | Diabetes, NIDDM^c^ |
| *GCLC* | R127C | N | C | Gamma-Etamylcysteine synthetase deficiency |
| *GFAP* | R88C | Mma/C | Mma | Alexander disease |
| *GFAP* | R88S | Mma/C | Mma | Alexander disease |
| *GFAP* | P47L | Mma/C | Mma | Alexander disease |
| *GJA8* | R23T | Gtm | PM | Cataract, congenital nuclear |
| *GJB1* | A39P | Gtm | PM | Charcot-Marie-Tooth disease 1 |
| *GJB1* | A40T | Gtm | PM | Charcot-Marie-Tooth disease |
| *GJB1* | R22G | Gtm | PM | Charcot-Marie-Tooth disease |
| *GJB1* | N205I | Gtm | PM | Charcot-Marie-Tooth disease 2 with deafness |
| *GJB1* | N205S | Gtm | PM | Charcot-Marie-Tooth disease |
| *GJB1* | E208G | Gtm | PM | Charcot-Marie-Tooth disease |
| *GJB1* | E41K | Gtm | Mtm/Gtm | Charcot-Marie-Tooth disease |
| *GJB1* | G21D | Gtm | PM | Charcot-Marie-Tooth disease |
| *GJB1* | I20S | Gtm | Mtm/PM | Charcot-Marie-Tooth disease |
| *GJB1* | I28N | Gtm | Mtm/PM | Charcot-Marie-Tooth disease |
| *GJB1* | I28T | Gtm | Mtm/PM | Charcot-Marie-Tooth disease |
| *GJB1* | I30N | Gtm | Mtm/PM | Charcot-Marie-Tooth disease |
| *GJB1* | I30T | Gtm | Mtm/PM | Charcot-Marie-Tooth disease |
| *GJB1* | L25P | Gtm | Mtm/PM | Charcot-Marie-Tooth disease 1 |
| *GJB1* | M194V | Gtm | PM | Charcot-Marie-Tooth disease |
| *GJB1* | M34K | Gtm | PM | Charcot-Marie-Tooth disease 1 |
| *GJB1* | M34T | Gtm | PM | Charcot-Marie-Tooth disease |
| *GJB1* | W24C | Gtm | PM | Charcot-Marie-Tooth disease |
| *GJB1* | W3R | Gtm | Mtm/Gtm | Charcot-Marie-Tooth disease |
| *GJB1* | W3S | Gtm | Mtm/Gtm | Charcot-Marie-Tooth disease |
| *GJB1* | V23A | Gtm | PM | Charcot-Marie-Tooth disease |
| *GJB1* | V23E | Gtm | PM | Charcot-Marie-Tooth disease |
| *GJB1* | V35M | Gtm | PM | Charcot-Marie-Tooth disease |
| *GJB1* | V37M | Gtm | PM | Charcot-Marie-Tooth disease |
| *GJB1* | V38M | Gtm | PM | Charcot-Marie-Tooth disease |
| *GJB2* | R32C | Gtm | PM | Deafness, autosomal recessive 1 |
| *GJB2* | R32H | Gtm | PM | Deafness |
| *GJB2* | R32L | Gtm | PM | Deafness |
| *GJB2* | I35S | Gtm | PM | Deafness |
| *GJB2* | S19T | Gtm | PM | Deafness, autosomal recessive 1 |
| *GNE* | C13S | C | Mma/C | Myopathy, distal, with rimmed vacuoles |
| *GNE* | P36L | C | Mma/C | Inclusion body myopathy |
| *GPI* | R83W | C | Mma/C | Ecosephosphate isomerase deficiency |
| *GPR143* | R5C | Mtm | Gtm | Albinism, ocular |
| *GUCY2D* | M1I | Mtm/PM | PM | Leber congenital amaurosis |
| *GUCY2D* | M1I | Mtm/PM | PM | Leber congenital amaurosis |
| *GUCY2D* | M1K | Mtm/PM | PM | Leber congenital amaurosis |
| *HESX1* | R160C | N | C | Septo-optic dysplasia |
| *HLXB9* | R293W | N | C | Currarino syndrome |
| *HLXB9* | R294Q | N | C | Currarino syndrome |
| *HLXB9* | R294W | N | C | Currarino syndrome |
| *HSD17B3* | P282L | S | Gtm | Pseudohermaphroditism |
| *HSD17B4* | G16S | Mma/P | Mma | D-bifunctionalPtein deficiency |
| *IL2RG* | C278W | PM | Gtm | Immunodeficiency, severe combined |
| *IL2RG* | G271E | PM | Gtm | Immunodeficiency, severe combined |
| *IL2RG* | M270R | PM | S | Immunodeficiency, severe combined |
| *IPF1* | R197H | N | C | Diabetes mellitus, type 2 |
| *IRF6* | R6C | Mma | Mma/C | Van derWoude syndrome |
| *KCNA1* | I177N | PM | Gtm | Episodic ataxia / myokymia |
| *KCNA1* | L329I | PM | Gtm | Episodic ataxia |
| *KCNA1* | F184C | PM | Gtm | Episodic ataxia / myokymia |
| *KCNA1* | S342I | PM | Gtm | Episodic ataxia |
| *KCNE2* | M54T | Gtm | PM | Cardiac arrhythmia |
| *KCNJ11* | L147P | PM | Gtm | Hypoglycaemia, persistent hyperinsulinaemic |
| *KCNJ1* | F95S | Mtm/Gtm | MtmPM | Bartter syndrome |
| *KCNJ1* | W99C | Mtm/Gtm | MtmPM | Bartter syndrome |
| *LCAT* | N5I | S | Gtm | Lecithin:cholesterol acyltransferase deficiency |
| *LDLR* | I771F | PM | Gtm | Hypercholesterolaemia |
| *LHCGR* | C543R | Gtm | PM | Pseudohermaphroditism |
| *LMNA* | R25G | Mma | Mma/C | Muscular dystrophy,Emery-Dreifuss |
| *LMNA* | R25P | Mma | Mma/C | Muscular dystrophy,Emery-Dreifuss |
| *MLC1* | N141S | Gtm | PM | MegalencephalicLkoencephalopathy |
| *MTM1* | P226T | P | C | Myotubular myopathy |
| *MTM1* | Y68D | P | C | Myotubular myopathy |
| *NEFL* | E7K | C | Mma | Charcot-Marie-Tooth disease 2 |
| *NEFL* | P8R | C | Mma | Charcot-Marie-Tooth disease 2 |
| *NIPBL* | A1246G | PM | N | Cornelia deLange syndrome |
| *NIPBL* | A2390T | PM | N | Cornelia deLange syndrome |
| *NIPBL* | R1789L | PM | N | Cornelia deLange syndrome |
| *NIPBL* | R1828Q | PM | N | Cornelia deLange syndrome |
| *NIPBL* | R1856T | PM | N | Cornelia deLange syndrome |
| *NIPBL* | R2298C | PM | N | Cornelia deLange syndrome |
| *NIPBL* | R2298H | PM | N | Cornelia deLange syndrome |
| *NIPBL* | N2236I | PM | N | Cornelia deLange syndrome |
| *NIPBL* | D1803V | PM | N | Cornelia deLange syndrome |
| *NIPBL* | C1311R | PM | N | Cornelia deLange syndrome |
| *NIPBL* | G2312R | PM | N | Cornelia deLange syndrome |
| *NIPBL* | G2381A | PM | N | Cornelia deLange syndrome |
| *NIPBL* | L1312P | PM | N | Cornelia deLange syndrome |
| *NIPBL* | L1348R | PM | N | Cornelia deLange syndrome |
| *NIPBL* | L2092V | PM | N | Cornelia deLange syndrome |
| *NIPBL* | M1K | PM | N | Cornelia deLange syndrome |
| *NIPBL* | Y2430C | PM | N | Cornelia deLange syndrome |
| *NIPBL* | Y2440H | PM | N | Cornelia deLange syndrome |
| *NPC1* | M272R | Gtm | PM | Niemann-Pick disease C |
| *NPC1* | P691L | Gtm | PM | Niemann-Pick disease C |
| *NPHS1* | A1078D | PM | S | Congenital nephrotic syndrome, Finnish type |
| *OCA2* | W652R | PM | Gtm | Albinism, oculocutaneousII |
| *PAXM* | R56L | Mma/N | N | Waardenburg syndrome |
| *PAX6* | N17S | N | Mma/N | Aniridia |
| *PCCA* | R52W | Mma | MmaC | Propionic acidaemia |
| *PKHD1* | W3871R | Gtm | S | PolyCtic kidney disease |
| *PLN* | R9C | Mtm/PM | PM | Cardiomyopathy, dilated |
| *PLP1* | A242E | PM | Gtm | Pelizaeus-Merzbacher disease |
| *PLP1* | G245E | PM | Gtm | Pelizaeus-Merzbacher disease |
| *PPOX* | H20P | Mma/C | Mma/S | Porphyria, variegate |
| *PPOX* | I12T | Mma/C | Mma | Porphyria, variegate |
| *PPOX* | M1L | Mma/C | Mma/S | Porphyria, variegate |
| *PRNP* | M232R | PM | Gtm | Creutzfeldt-Jakob syndrome |
| *PP1* | R120C | N | C | Pituitary hormone deficiency |
| *PP1* | R120H | N | C | Pituitary hormone deficiency |
| *PSEN1* | H163R | Gtm | PM | Alzheimer disease |
| *PSEN1* | L166R | Gtm | PM | Alzheimer disease |
| *PSEN1* | L166P | Gtm | PM | Alzheimer disease |
| *PSEN1* | L171P | Gtm | PM | Alzheimer disease |
| *PSEN1* | L174M | Gtm | PM | Alzheimer disease |
| *PSEN1* | M139I | Gtm | PM | Alzheimer disease |
| *PSEN1* | M146I | Gtm | PM | Alzheimer disease |
| *PSEN1* | M146I | Gtm | PM | Alzheimer disease |
| *PSEN1* | M146I | Gtm | PM | Alzheimer disease |
| *PSEN1* | F175S | Gtm | PM | Alzheimer disease |
| *PSEN1* | F177S | Gtm | PM | Alzheimer disease |
| *PSEN1* | S169P | Gtm | PM | Alzheimer disease |
| *PSEN1* | S178P | Gtm | PM | Alzheimer disease |
| *PSEN1* | T147I | Gtm | PM | Alzheimer disease |
| *PSEN1* | W165C | Gtm | PM | Alzheimer disease |
| *PSEN1* | W165G | Gtm | PM | Alzheimer disease |
| *PTH* | S23P | S | C | Hypoparathyroidism |
| *RAX* | R192Q | N | C | Anophthalmia and sclerocornea |
| *RHD* | G339E | PM | Gtm | Reduced expression (weak D) |
| *RHD* | S333N | PM | Gtm | RhDblood group variant |
| *SDHD* | D92Y | Mma | Mtm | Paraganglioma |
| *SHOX* | R168L | N | C | Short stature |
| *SHOX* | R168W | N | C | Leri-Weill dyschondrosteosis |
| *SHOX* | R173C | N | C | Leri-Weill dyschondrosteosis |
| *SHOX* | R173H | N | C | Leri-Weill dyschondrosteosis |
| *SLC25A20* | G81R | PM | Gtm | Carnitine-acylcarnitine carrier deficiency |
| *SMPD1* | D49V | Mma/PM | Mma/Gtm | Niemann-Pick disease |
| *SMPD1* | M1R | Mma/PM | Mtm/PM | Niemann-Pick disease |
| *TMC1* | M654V | Gtm | PM | Deafness |
| *UCHL1* | I82M | MmaC | MpsC | Parkinson disease |
| *UCHL1* | S7Y | MmaC | C | Parkinson disease, lower risk, association with |
| *VDR* | R50Q | N | C | Rickets, vitamin D resistant |
| *VMD2* | R13H | Mtm/Gtm | Gtm | Best macular dystrophy |
| *VMD2* | R25Q | Mtm/Gtm | Gtm | Best macular dystrophy |
| *VMD2* | R25W | Mtm/Gtm | Gtm | Best macular dystrophy |
| *VMD2* | D104H | Mtm/Gtm | Mma/Gtm | Best macular dystrophy |
| *VMD2* | G26R | Mtm/Gtm | Mtm | Best macular dystrophy |
| *VMD2* | K30R | Mtm/Gtm | Mtm | Best macular dystrophy |
| *VMD2* | T6R | Mtm/Gtm | Mtm | Best macular dystrophy |
| *VMD2* | Y85H | Mtm/Gtm | Mma/Gtm | Best macular dystrophy |
| *XRCC1* | R399Q | N | P | Increased lung cancer risk, association with |

^a^autoimmune polyendocrinopathy, candidiasis, and ectodermal dystrophy

^b^ectonucleotide pyrophosphatase/phosphodiesterase

^c^non–insulin-dependent diabetes mellitus
