# Supplementary material for: Prediction of disease-related mutations affecting protein localization
Source: BMC Genomics. 2009 Mar 23;10:122. doi: 10.1186/1471-2164-10-122 (PMC2680896; doi:10.1186/1471-2164-10-122)
Supplement: Additional File 6 — Mutations predicted by WolF PSORT to alter protein localization. List of disease-causing mutations predicted to be related to protein localization by WoLF PSORT. [file 1471-2164-10-122-S6.doc]

## Additional file 6 - Mutations predicted by WoLF PSORT to alter protein localization

| Gene | Amino acid change | Wildtype localization | Mutant localization | Disease |
| --- | --- | --- | --- | --- |
| *ABCB7* | I400M | PM | M | Sideroblastic anaemia and ataxia |
| *ABCD1* | T254K | PM | M | Adrenoleukodystrophy |
| *ABCD1* | W339R | PM | M | Adrenomyeloneuropathy |
| *ABCD1* | S342P | PM | M | Adrenoleukodystrophy |
| *ABCD1* | G343D | PM | M | Adrenoleukodystrophy |
| *AGA* | L15R | S | M | Aspartylglucosaminuria |
| *AGXT* | G41R | C | M | Hyperoxaluria |
| *AICDA* | R24W | N | S | Hyper-IgM syndrome |
| *AIRE* | L28P | S | M | APECED^a^ |
| *AIRE* | L29P | S | M | APECED |
| *ALDH3A2* | P114L | C | PM | Sjoegren-Larsson syndrome |
| *ALDH3A2* | P121L | C | PM | Sjoegren-Larsson syndrome |
| *ARSB* | R315Q | G/ER_G | L | Mucopolysaccharidosis VI |
| *ASAH1* | E138V | S | ER | Farber disease |
| *BBS1* | H35R | C/C_N | N | Bardet-Biedl syndrome |
| *BCS1L* | S78G | C_N | S | GRACILE syndrome |
| *BCS1L* | S277N | C_N | S | Complex 3 deficiency |
| *BSG* | E926K | S/S_PM | ER | Blood group variation |
| *BTK* | K17E | M | N/C_N | Agammaglobulinaemia |
| *BTK* | K19E | M | N/C_N | Agammaglobulinaemia |
| *CBS* | V454E | PM | C | Homocystinuria |
| *CBS* | L456P | PM | C | Homocystinuria |
| *CBS* | V534D | PM | C | Homocystinuria |
| *CBS* | L539S | PM | C | Homocystinuria |
| *CD40LG* | M36R | S | M | Hyper-IgM syndrome |
| *CD40LG* | G38R | S | M | Hyper-IgM syndrome |
| *CHL1* | L17F | PM | P | Schizophrenia, association with |
| *CNGB3* | P309L | C | PM | Achromatopsia |
| *COL10A1* | G18E | ER | S | meta physeal chondrodysplasia, Schmid |
| *COL10A1* | G18R | ER | M | meta physeal chondrodysplasia, Schmid |
| *CPT1A* | R123C | C | PM | Carnitine palmitoyltransferase 1 deficiency |
| *CYP19A1* | W39R | ER | C | Breast cancer, decreased risk, association with |
| *EDA* | L55R | C | N | Ectodermal dysplasia |
| *EIF2B5* | A16D | C_N | M | Leukoencephalopathy with vanishing white matter |
| *EIF2B5* | I385V | C_N | M | Leukoencephalopathy with vanishing white matter |
| *EIF2B5* | L425R | C_N | N | Leukoencephalopathy with vanishing white matter |
| *EIF2B5* | F56V | C_N | M_N | Leukoencephalopathy with vanishing white matter |
| *EIF2B5* | P323S | C_N | M_N | Leukoencephalopathy with vanishing white matter |
| *EIF2B5* | E81K | C_N | C_M/M_N | Leukoencephalopathy with vanishing white matter |
| *EIF2B5* | E198K | C_N | C_M/M_N | Leukoencephalopathy with vanishing white matter |
| *EIF2B5* | E650K | C_N | C_M/M_N | Leukoencephalopathy with vanishing white matter |
| *EMD* | M1T | M/P/M_P | N/C/C_N | Muscular dystrophy, Emery-Dreifuss |
| *ENG* | V311G | PM | S | Haemorrhagic telangiectasia 1 |
| *ERCC2* | D234N | N | C | Xeroderma pigmentosum |
| *EYA1* | L472R | C | N | Branchio-oto-renal syndrome |
| *F8* | S-1R | S | M | Haemophilia A |
| *FAH* | N16I | C | cysk | Tyrosinaemia 1 |
| *FANCG* | R22P | M | N | Fanconi anaemia |
| *FANCG* | L71P | M | N | Fanconi anaemia |
| *FBN1* | I1909T | N | S | Marfan syndrome |
| *FGFR2* | C278Y | ER | PM | Crouzon syndrome |
| *FGFR2* | C278F | ER | PM | Crouzon syndrome |
| *FGFR2* | C342S | ER | PM | Crouzon syndrome |
| *FGFR2* | C342R | ER | PM | Crouzon syndrome |
| *FGFR2* | C342G | ER | PM | Pfeiffer syndrome |
| *FGFR2* | C342Y | ER | PM | Crouzon syndrome |
| *FGFR2* | C342S | ER | PM | Jackson-Weiss syndrome |
| *FGFR2* | C342F | ER | PM | Crouzon syndrome |
| *FGFR2* | C342W | ER | PM | Crouzon syndrome |
| *FGFR2* | G384R | ER | PM | Craniosynostosis |
| *FLNB* | G1586R | C_N | N | Larsen syndrome |
| *GALNS* | G23R | S | M | Mucopolysaccharidosis IVa |
| *GCK* | Q138P | C | N | Diabetes, MODY^b^ |
| *GCK* | G193R | C | C_N | Diabetes, MODY |
| *GNAS* | F246S | C | C_N | Albright hereditary osteodystrophy |
| *GUSB* | Y320C | N | S | Mucopolysaccharidosis VII |
| *GUSB* | R374C | N | S | Mucopolysaccharidosis VII |
| *GUSB* | R382C | N | S | Mucopolysaccharidosis VII |
| *GUSB* | Y495C | N | S | Mucopolysaccharidosis VII |
| *GUSB* | Y508C | N | S | Mucopolysaccharidosis VII |
| *GUSB* | R577L | N | S | Mucopolysaccharidosis VII |
| *GUSB* | W627C | N | S | Mucopolysaccharidosis VII |
| *HLCS* | G518E | C | N | Multiple carboxylase deficiency |
| *HLXB9* | W289L | N | C_N | Currarino syndrome |
| *HLXB9* | W289G | N | C_N | Currarino syndrome |
| *HSD17B3* | P282L | S | PM | Pseudohermaphroditism |
| *HSD17B4* | G16S | C | C_M | D-bifunctional protein deficiency |
| *HSD3B2* | A82T | PM | C | Pseudohermaphroditism |
| *HSD3B7* | E147K | E.R_M | C | 3 beta-hydroxysteroid oxidoreductase deficiency |
| *IL12RB1* | Q214R | PM | S | Mycobacterial infection |
| *KCNE2* | T8A | C | PM | Long QT interval, drug induced, association with |
| *KCNE2* | Q9E | C | PM | Cardiac arrhythmia |
| *KCNJ1* | F95S | PM | N | Bartter syndrome |
| *LDHB* | A35E | C | cysk | Lactate dehydrogenase deficiency |
| *MAN2B1* | E402K | S | L | Mannosidosis, alpha |
| *MEN1* | D153V | M | PM | Hyperparathyroidism |
| *MEN1* | S154I | M | PM | Multiple endocrine neoplasia 1 |
| *MRAP* | M1I | PM | C | Glucocorticoid deficiency 2 |
| *MSH2* | G751R | N | C | Colorectal cancer, non-polyposis |
| *MTHFR* | I153M | C_N | N | Homocystinuria |
| *MTHFR* | I226T | C_N | N | Reduced homocysteine levels, association with |
| *MTHFR* | N324S | C_N | N | Methylenetetrahydrofolate reductase deficiency |
| *MTHFR* | R357C | C_N | C | Homocystinuria |
| *MTRR* | R3W | N | C | Homocystinuria |
| *MTRR* | M22I | N | M | Coronary artery disease, association with |
| *MTRR* | C405R | N | M | Methionine synthase reductase deficiency |
| *MUT* | R369C | M | C | Methylmalonic aciduria |
| *MUT* | Y587C | M | C | Methylmalonic aciduria |
| *MUT* | L617R | M | C | Methylmalonic aciduria |
| *MUT* | K621N | M | C | Methylmalonic aciduria |
| *MUT* | G626C | M | C | Methylmalonic aciduria |
| *MVK* | S150L | S | ER_M | Hyperimmunoglobulin D and periodic fever syndrome |
| *MYH9* | I1816V | C_N | N | Alport syndrome with macrothrombocytopaenia |
| *MYL2* | A13T | C | N | Cardiomyopathy, hypertrophic |
| *MYL2* | F18L | C | M | Cardiomyopathy, hypertrophic |
| *NCF1* | G262S | C | N | Chronic granulomatous disease |
| *NPR2* | R957C | ER | PM | Acromesomelic dysplasia, |
| *OTC* | R26Q | M | C | Ornithine transcarbamylase deficiency |
| *PCBD1* | E58K | S | C_N | Hyperphenylalaninaemia |
| *PCBD1* | T79I | S | C_N | Hyperphenylalaninaemia |
| *PCBD1* | C82R | S | C_N | Hyperphenylalaninaemia |
| *PCBD1* | E97K | S | C_N | Hyperphenylalaninaemia |
| *PEX10* | H290Q | N | C | Neonatal adrenoleukodystrophy |
| *PEX12* | S320F | C_N | N | Peroxisome biogenesis disorder 3 |
| *PON2* | S311C | S | ER | Coronary artery disease, lower risk, association |
| *PPOX* | I12T | S | M | Porphyria, variegate |
| *PTGS1* | P17L | S | L | Inhibition of prostaglandin H2 formation |
| *PTH* | S23P | N | S | Hypoparathyroidism |
| *PTPN11* | G464A | C | C_M | ML^c^ / LEOPARD syndrome |
| *PTPN11* | T468M | C | C_M | ML / LEOPARD syndrome |
| *PXMP3* | C247R | N | PM | Peroxisome biogenesis disorder |
| *QDPR* | G17R | C | M | Dihydropteridine reductase deficiency |
| *QPDR* | G17V | C | M | Dihydropteridine reductasedeficiency |
| *QPDR* | W36R | C | M | Dihydropteridine reductase deficiency |
| *RAG2* | F206C | C_N | C | Omenn syndrome |
| *RB1* | P20L | N | M | Retinoblastoma |
| *RPE65* | G40S | C | C_N | Retinitis pigmentosa |
| *RPE65* | R44Q | C | C_N | Leber congenital amaurosis |
| *RPE65* | E102K | C | C_N | Retinitis pigmentosa |
| *RPE65* | N321K | C | C_N | Leber congenital amaurosis |
| *RPE65* | C330Y | C | C_N | Retinitis pigmentosa |
| *RPE65* | L341S | C | N/C_N | Retinitis pigmentosa |
| *SDHD* | R70G | M | S | Paraganglioma |
| *SDHD* | P81L | M | S | Paraganglioma |
| *SDHD* | L139P | M | S | Paraganglioma |
| *SERPINC1* | L-10P | S | M | Antithrombin deficiency |
| *SERPING1* | P149L | S | ER | Angioneurotic oedema |
| *SERPING1* | R444C | S | ER | Angioneurotic oedema |
| *SERPING1* | R444L | S | ER | Angioneurotic oedema |
| *SH2D1A* | Y7C | C | S | Lymphoproliferative syndrome, X-linked |
| *SH2D1A* | Y54C | C | S | Lymphoproliferative syndrome, X-linked |
| *SHOX* | L154P | N | C | Leri-Weill dyschondrosteosis |
| *SHOX* | V161A | N | C | Leri-Weill dyschondrosteosis |
| *SHOX* | R173C | N | C | Leri-Weill dyschondrosteosis |
| *SHOX* | R173H | N | C | Leri-Weill dyschondrosteosis |
| *SLC25A15* | G27R | C | S | HHH^d^ syndrome |
| *SMN1* | A2G | N | M | Spinal muscular atrophy |
| *SMPD1* | D49V | PM | N | Niemann-Pick disease |
| *SPG7* | A10S | PM | M | Spastic paraplegia |
| *TH* | L205P | C_N | N | Parkinsonism, L-DOPA-responsive |
| *TIMM8A* | C66W | S | N | Mohr-Tranebjaerg syndrome |
| *TNNT2* | F110I | N | C_N | Cardiomyopathy, hypertrophic |
| *TNNT2* | N271I | N | C_N | Cardiomyopathy, hypertrophic |
| *TPI1* | M-1K | C | M | Triosephosphate isomerase deficiency |
| *TYR* | M1V | PM | S_PM | Albinism, oculocutaneous 1 |
| *UCP3* | V102I | C_M | C | Obesity, severe, with diabetes |
| *UGT1A1* | L175Q | PM | ER | Crigler-Najjar syndrome 2 |
| *UGT1A3* | Q6R | ER | P | Enzyme activity, association with |
| *UGT1A3* | W11R | ER | P | Enzyme activity, association with |
| *UROD* | G25E | C | C_N | Porphyria, cutanea tarda |
| *UROS* | Y19C | C | C_N | Porphyria, erythropoietic |
| *WAS* | T48I | N | C | Wiskott-Aldrich syndrome |
| *WAS* | P58L | N | C | Wiskott-Aldrich syndrome |
| *VDR* | H35Q | N | C_N | Rickets, vitamin D resistant |
| *VDR* | F47I | N | C_N | Rickets, vitamin D resistant |
| *VDR* | R50Q | N | C_N | Rickets, vitamin D resistant |
| *VHL* | F76I | C_N | C | Von Hippel-Lindau syndrome |
| *VHL* | F76S | C_N | C | Von Hippel-Lindau syndrome |
| *VHL* | N78S | C_N | C | Von Hippel-Lindau syndrome |
| *VHL* | S80I | C_N | C | Von Hippel-Lindau syndrome |
| *VHL* | P81S | C_N | C | Von Hippel-Lindau syndrome |
| *VHL* | P86L | C_N | C | Von Hippel-Lindau syndrome |
| *VHL* | P86S | C_N | C | Von Hippel-Lindau syndrome |
| *VHL* | W88S | C_N | C | Von Hippel-Lindau syndrome |
| *VHL* | N90I | C_N | C | Von Hippel-Lindau syndrome |
| *VHL* | G93S | C_N | C | Von Hippel-Lindau syndrome |
| *VHL* | F119S | C_N | C | Phaeochromocytoma |
| *VHL* | N131S | C_N | C | Von Hippel-Lindau syndrome |
| *VHL* | F136S | C_N | C | Von Hippel-Lindau syndrome |
| *VHL* | A149S | C_N | C | Von Hippel-Lindau syndrome |
| *VHL* | P154L | C_N | C | Von Hippel-Lindau syndrome |
| *VHL* | T157I | C_N | C | Von Hippel-Lindau syndrome |
| *VHL* | P192S | C_N | C | Polycythaemia, with high epo concentration |

^a^autoimmune polyendocrinopathy, candidiasis, and ectodermal dystrophy

^b^maturity onset diabetes of the young

^c^multiple-lentigines

^d^hyperornithinemia-hyperammonemia-homocitrullinemia
