# Supplementary material for: Prediction of disease-related mutations affecting protein localization
Source: BMC Genomics. 2009 Mar 23;10:122. doi: 10.1186/1471-2164-10-122 (PMC2680896; doi:10.1186/1471-2164-10-122)
Supplement: Additional File 7 — Comparison of observed and expected numbers of mutations in the dataset. Statistical analysis of numbers of mutations. [file 1471-2164-10-122-S7.doc]

## Additional file 7 - Comparison of observed and expected numbers of mutations in the dataset.^a^

Amino acid Observed Expected χ^2^ p-value

A 1 292 1 567 *48.3* 3.7E-12***

C 1 389 520 **1452.2** 0***

D 1 077 1 056 0.4 5.2E-1

E 990 1 540 *196.4* 0***

F 577 852 *88.8* 0***

G 2 793 1 484 **1155.5** 0***

H 575 583 0.1 7.4E-1

I 786 995 *43.9* 3.5E-11***

K 462 1 265 *509.7* 0***

L 1 627 2 254 *174.4* 0***

M 725 496 **105.7** 0***

N 668 810 *24.9* 6.1E-7

P 1 188 1 376 *25.7* 4.0E-7***

Q 457 1 045 *330.9* 0***

R 3 459 1 275 **3741.1** 0***

S 1 153 1 823 *246.2* 0***

T 920 1 194 *62.9* 2.2E-15***

V 1 120 1 368 *45.0* 2.0E-11***

W 469 297 **99.6** 0***

Y 690 617 **8.6** 3.3E-3***

^a^Significance is marked as follows, *, p<0.05: **, p<0.01; ***, p<0.001.

Under representation of mutations is shown in italics and over representation in bold face.
