# Supplementary material for: Prediction of disease-related mutations affecting protein localization
Source: BMC Genomics. 2009 Mar 23;10:122. doi: 10.1186/1471-2164-10-122 (PMC2680896; doi:10.1186/1471-2164-10-122)
Supplement: Additional File 9 — Amino acid distribution of localization mutations predicted with WolF PSORT. Statistics of amino acid changes in the disease-causing mutations predicted to change localization by WoLF PSORT. [file 1471-2164-10-122-S9.doc]

## Additional file 9 -Amino acid distribution of localization mutations predicted with WoLF PSORT

Mutant

| Wild type |  | C | D | E | F | G | H | I | K | L | M | N | P | Q | R | S | T | V | W | Y | Total |
| --- | --- | --- | --- | --- | --- | --- | --- | --- | --- | --- | --- | --- | --- | --- | --- | --- | --- | --- | --- | --- | --- |
| A |  |  | 1 | 1 |  | 1 |  |  |  |  |  |  |  |  |  | 2 | 2 |  |  |  | 7 |
| C |  |  |  |  | 2 | 1 |  |  |  |  |  |  |  |  | 4 | 2 |  |  | 2 | 3 | 14 |
| D |  |  |  |  |  |  |  |  |  |  |  | 1 |  |  |  |  |  | 2 |  |  | 3 |
| E |  |  |  |  |  |  |  |  | 9 |  |  |  |  |  |  |  |  | 1 |  |  | 10 |
| F |  | 1 |  |  |  |  |  | 3 |  | 1 |  |  |  |  |  | 5 |  | 1 |  |  | 11 |
| G | 1 | 1 | 1 | 3 |  |  |  |  |  |  |  |  |  |  | 10 | 4 |  | 1 |  |  | 21 |
| H |  |  |  |  |  |  |  |  |  |  |  |  |  | 2 | 1 |  |  |  |  |  | 3 |
| I |  |  |  |  |  |  |  |  |  |  | 2 |  |  |  |  |  | 3 | 2 |  |  | 7 |
| K |  |  |  | 2 |  |  |  |  |  |  |  | 1 |  |  |  |  |  |  |  |  | 3 |
| L |  |  |  |  | 1 |  |  |  |  |  |  |  | 8 | 1 | 5 | 2 |  |  |  |  | 17 |
| M |  |  |  |  |  |  |  | 2 | 1 |  |  |  |  |  | 1 |  | 1 | 1 |  |  | 6 |
| N |  |  |  |  |  |  |  | 3 | 1 |  |  |  |  |  |  | 3 |  |  |  |  | 7 |
| P |  |  |  |  |  |  |  |  |  | 11 |  |  |  |  |  | 4 |  |  |  |  | 15 |
| Q |  |  |  | 1 |  |  |  |  |  |  |  | 1 |  |  | 2 |  |  |  |  |  | 4 |
| R |  | 8 |  |  |  | 1 | 1 |  |  | 2 |  |  | 1 | 4 |  |  |  |  | 2 |  | 19 |
| S |  | 1 |  |  | 1 | 1 |  | 2 |  | 1 |  | 1 | 2 |  | 1 |  |  |  |  |  | 10 |
| T | 1 |  |  |  |  |  |  | 3 | 1 |  | 1 |  |  |  |  |  |  |  |  |  | 6 |
| V | 1 |  | 1 | 1 |  | 1 |  | 1 |  |  |  |  |  |  |  |  |  |  |  |  | 5 |
| W |  | 1 |  |  |  | 1 |  |  |  | 1 |  |  |  |  | 4 | 1 |  |  |  |  | 8 |
| Y |  | 7 |  |  |  |  |  |  |  |  |  |  |  |  |  |  |  |  |  |  | 7 |
| Total | 3 | 19 | 3 | 8 | 4 | 6 | 1 | 14 | 12 | 16 | 3 | 4 | 11 | 7 | 28 | 23 | 6 | 8 | 4 | 3 | 183 |
